# Supplementary material for: Human-like driving behaviour emerges from a risk-based driver model
Source: Nat Commun. 2020 Sep 29;11:4850. doi: 10.1038/s41467-020-18353-4 (PMC7525534; doi:10.1038/s41467-020-18353-4)
Supplement: Supplementary file 1 — Supplementary Information [file 41467_2020_18353_MOESM1_ESM.pdf]

Human-like driving behaviour emerges from a risk-based driver model
(Supplementary Information)

Kolekar et al.

Contents

- **Supplementary Figure 1:** This figure describes the boundary of the DRF modelled in this paper.
- **Supplementary Figure 2:** This figure describes the bouncing behaviour exhibited by satisficing controller.
- **Supplementary Figures 3-6:** These figures explain the calculations performed to arrive at the metrics used in Fig. 4 (Road scenarios) of the main text.
- **Supplementary Figures 7-9:** These figures explain the calculations performed to arrive at the metrics used in Fig. 5 (Traffic scenarios) of the main text.
- **Supplementary Tables 1-8:** These tables summarise the methods used in the literature used to validate our model.
- **Supplementary Notes:** These notes specify which data and which figures from the corresponding literature were used in the main text, especially in Figures 4 and 5.

Supplementary Figures

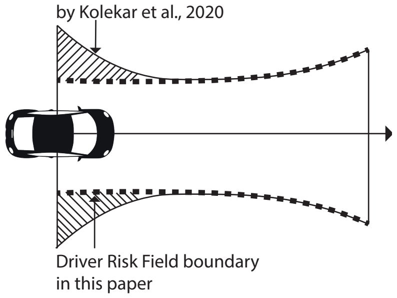

Supplementary Figure 1: Kolekar et al., (2020) [24] reported that the hatched region was only 'active' when the obstacle appeared instantaneously, or approached from the rear of the vehicle. In this paper, the DRF model focuses on previewed objects and hence the hatched region is neglected. This figure is reprinted from: Kolekar, S., De Winter, J. & Abbink, D. Which parts of the road guide obstacle avoidance? Quantifying the driver's risk field. *Applied Ergonomics* **89**, 103196 (2020), with permission from Elsevier

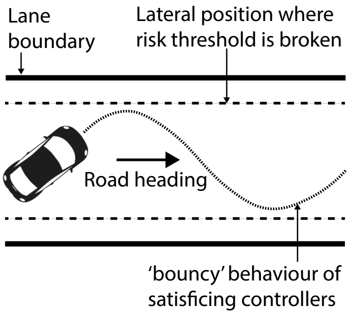

Supplementary Figure 2: 'Bouncing' behaviour exhibited by satisficing controllers since there is no correction until the risk threshold is crossed. The dashed line represents the lateral position at which the risk threshold is broken. That's when a correction (steering) is made.

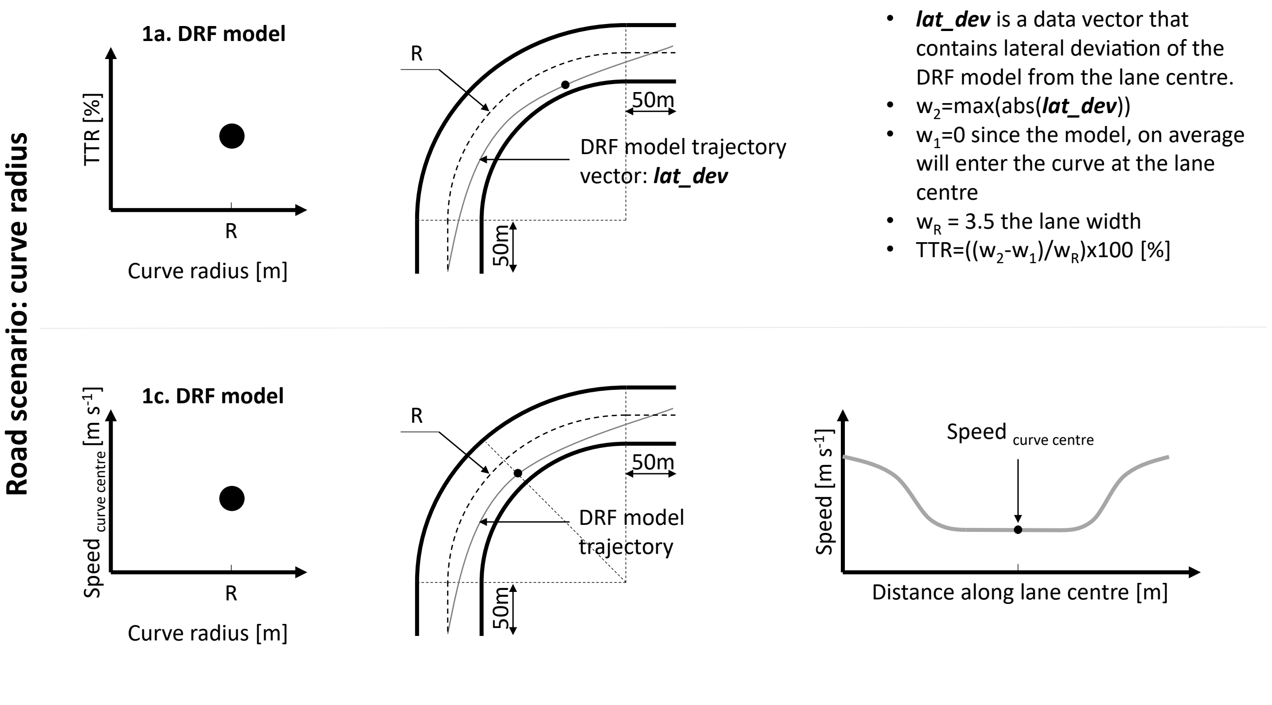

Supplementary Figure 3: This figure explains the calculations of the metrics used in the subfigures 1a and 1c in Fig. 4 of the main text.

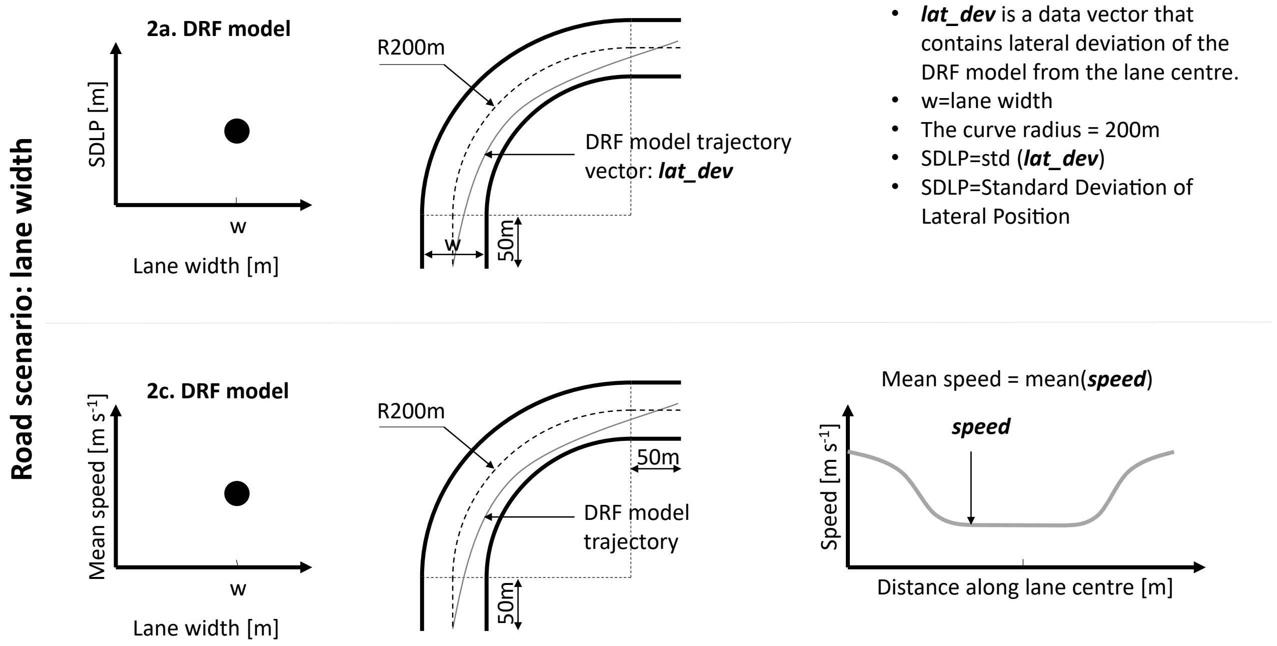

Supplementary Figure 4: This figure explains the calculations of the metrics used in the subfigures 2a and 2c in Fig. 4 of the main text.

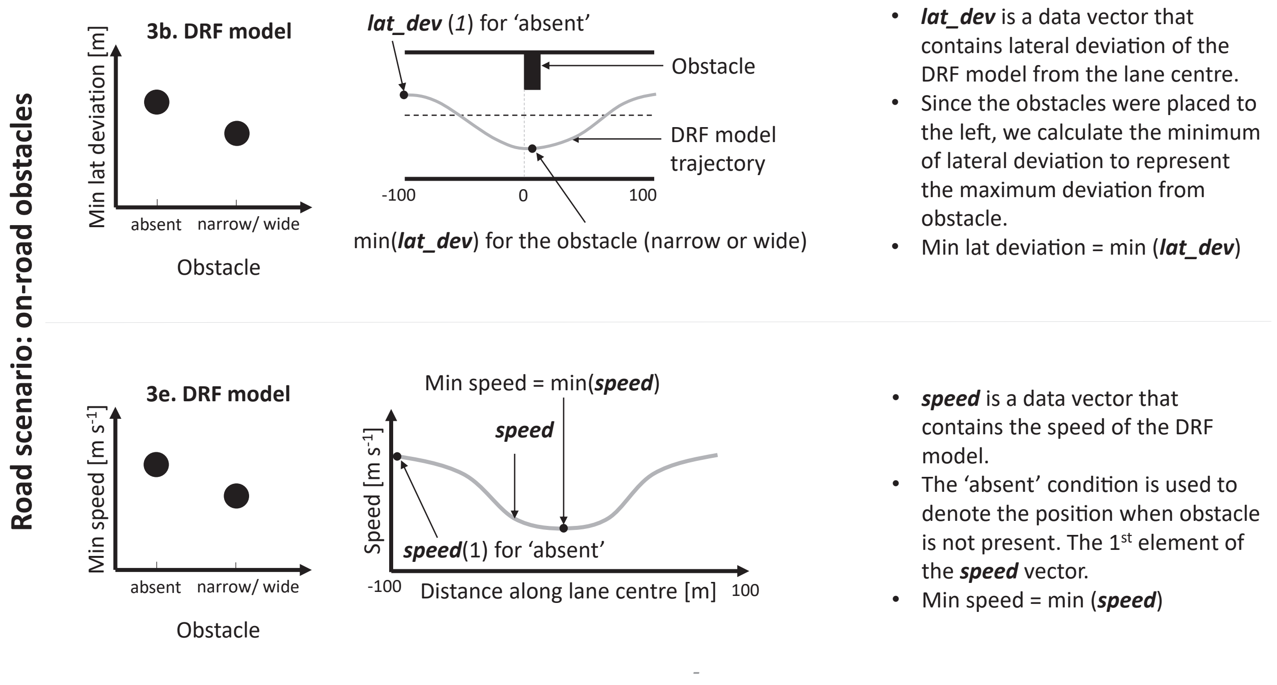

Supplementary Figure 5: This figure explains the calculations of the metrics used in the subfigures 3b and 3e in Fig. 4 of the main text.

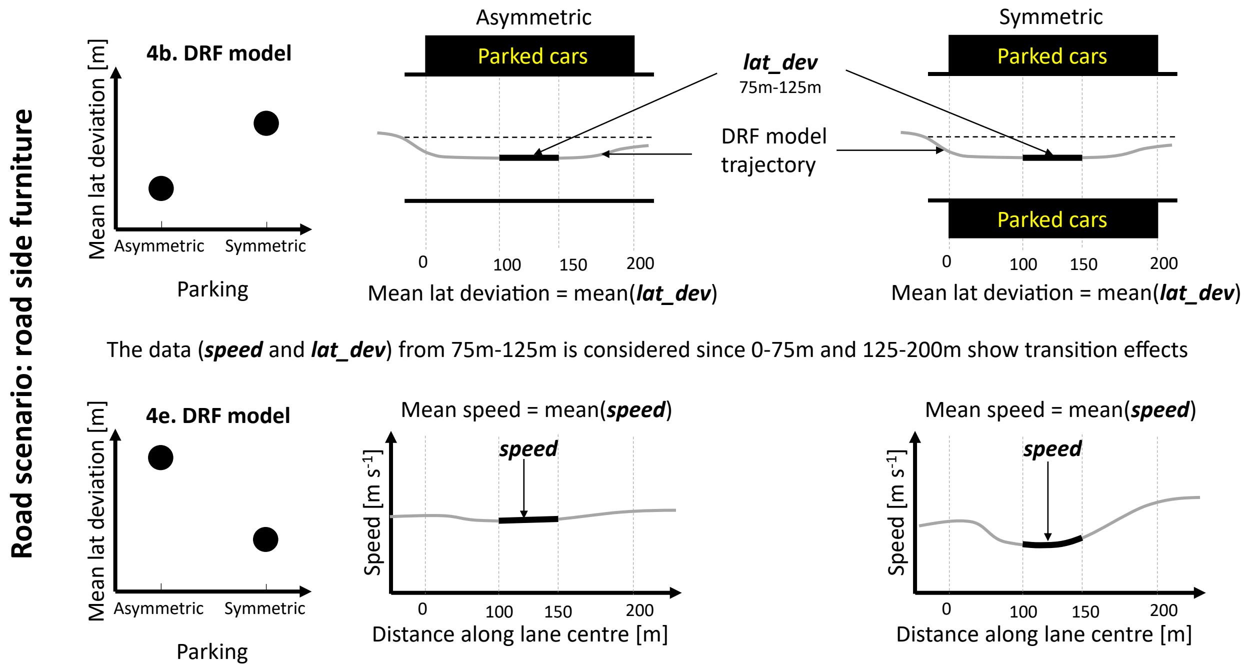

Supplementary Figure 6: This figure explains the calculations of the metrics used in the subfigures 4b and 4e in Fig. 4 of the main text.

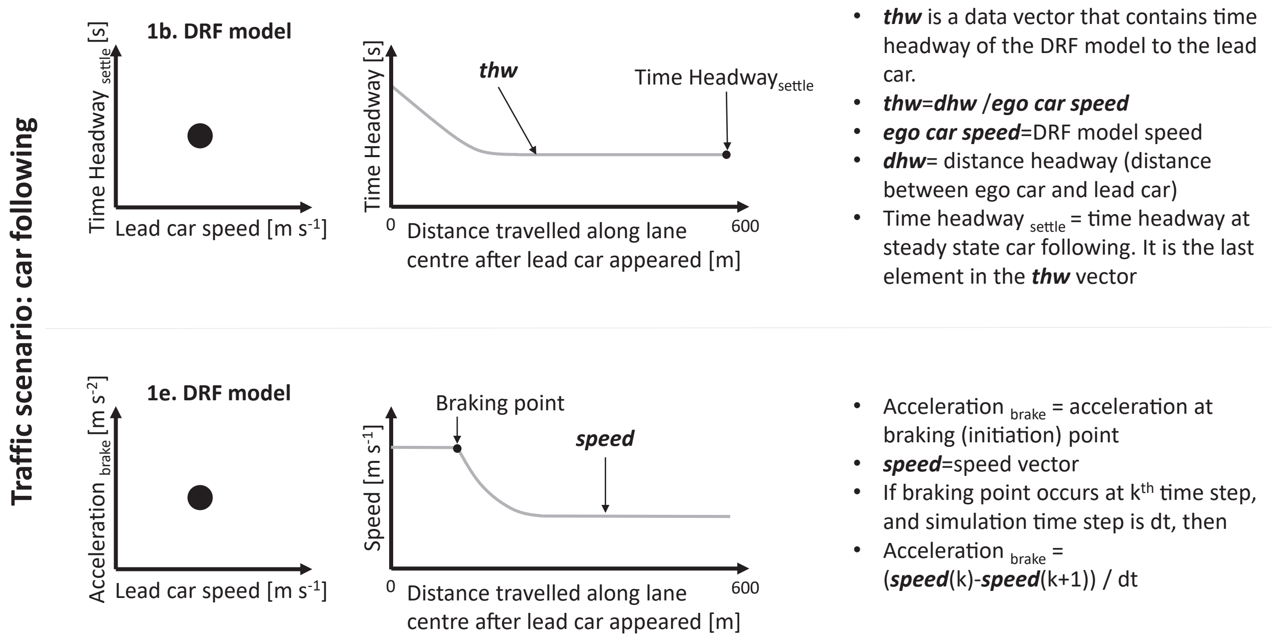

Supplementary Figure 7: This figure explains the calculations of the metrics used in the subfigures 1b and 1e in Fig. 5 of the main text.

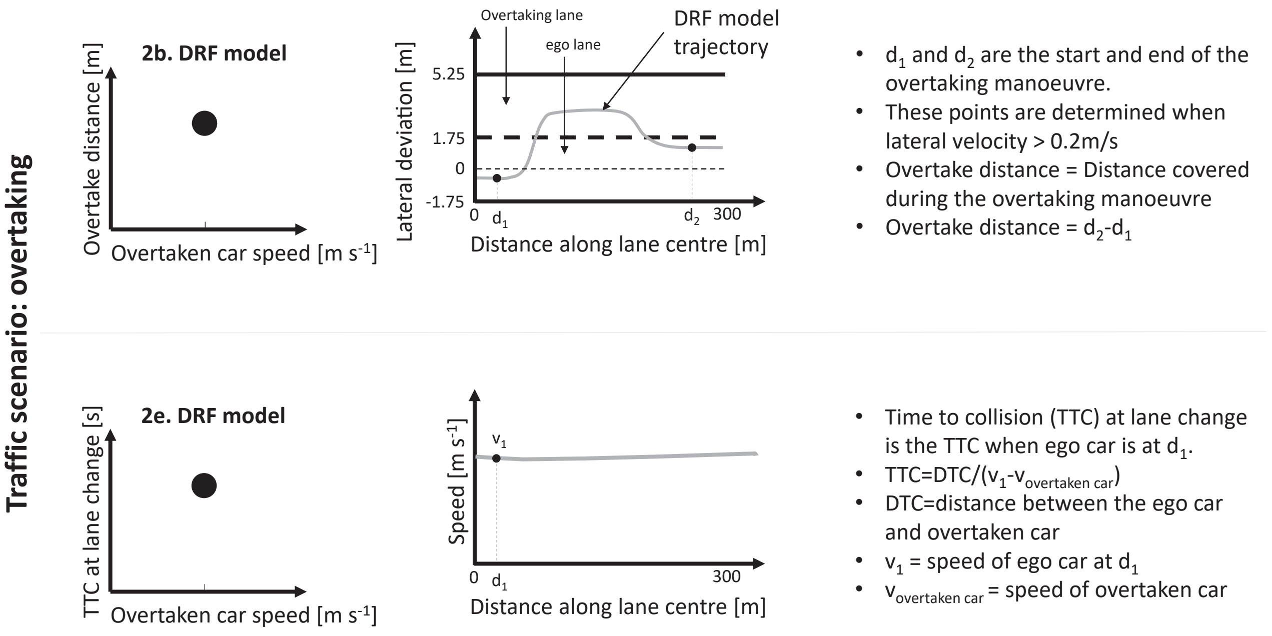

Supplementary Figure 8: This figure explains the calculations of the metrics used in the subfigures 2b and 2e in Fig. 5 of the main text.

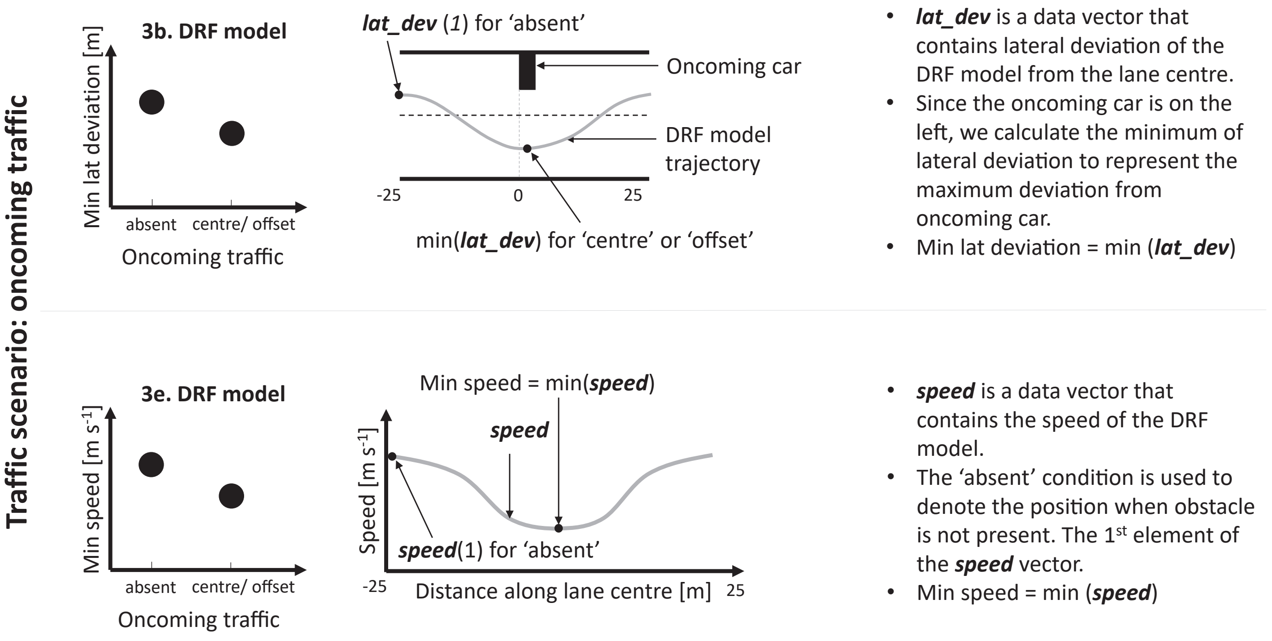

Supplementary Figure 9: This figure explains the calculations of the metrics used in the subfigures 3b and 3e in Fig. 5 of the main text.

Supplementary Tables

Validity for on-road behaviour: In the main text (Fig. 4 & 5) we selected certain studies from published literature to validate the predictions of the DRF model. We selected the studies based on the similarity of the conditions that they tested, to those simulated for the DRF model, so that the trends shown by the DRF model could be validated more closely. This meant that in some scenarios we chose studies performed in a simulator. In the following pages we give a brief description on the experimental apparatus so that the readers have a better understanding of the validity of the results. In scenarios where we used simulator based studies, we provide at least one on-road study which supports the findings of the simulator based study.

Supplementary Table. 1: Literature for metrics 1 and 2 of the road scenario-curve radius

		Paper	On-road/ Simulator	Description	Conclusion
1. Curve radius	The effect of curve radii on curve cutting behaviour (metric 1) was validated using the on-road study of Xu et al., 2018 [1]. The effect on speed (metric 2) was validated using the on-road study of Taragin and Leisch, 1954 [2].				
1. Curve radius	Metric 1	Xu et al., 2018 [1]	On-road	(N=4) Field driving experiments were performed on four two-lane mountain highways in China, and vehicle trajectories under natural driving conditions were acquired. The road lengths were 26.8 km, 45.2 km, 74.8 km, 121.9 km, and participants had to drive them on them twice. Cars used: Mitsubishi ASX, Buick Firstland GL8 Business, Mercedes Benz Vito Business	Curve cutting reduced as the curve radius increased (Main text: Fig. 4-1b)
1. Curve radius	Metric 2	Taragin and Leisch, 1954 [2]	On-road	(N=8400) The study was conducted on two-lane highways mainly in Maryland, New York, South Carolina, Illinois, and Minnesota. 8400 free moving passenger cars were observed on 35 different curves.	Speed increases with curve radius, and reaches an asymptote approaching straight road speed for a large radius (Main text: Fig. 4-1d)

Supplementary Table. 2: Literature for metric 1 of the road scenario-lane width

		Paper	On-road/ Simulator	Description	Conclusion
2. Lane width	The effect of lane width on standard deviation of lateral position (SDLP) was validated using the driving simulator based study of Godley et al., 2004 [3]. The simulator has a motion platform and has been validated for use in research. We also cite the on-road study of De Waard et al., 1995 [4] who found similar results that validate the DRF model's predictions. However, we used the simulator based study of Godley et al., 2004 since the conditions they used (lane widths) were similar to those simulated for the DRF model.				
2. Lane width	Metric 1	Godley et al., 2004 [3]	Simulator	(N=28) Experiment was conducted in the Monash University Accident Research Centre driving simulator, in Australia. This simulator has been validated for its use in examining road-based speeding countermeasures [5]. The driving simulator consisted of a Ford Falcon cabin. The simulator also contained a three-dimensional sound system reproducing tyre and engine noises, and a vertical motion platform providing road feel for accelerating, braking, cornering, and raised road objects.	Standard Deviation of Lateral Position (SDLP) which denotes the swerving behaviour of a car is reported to increase with lane width (Main text: Fig. 4-2b)
2. Lane width	Metric 1	De Waard et al., 1995 [4]	On-road	(N=28) The experiment was conducted on 12 km of woodland road and 10 km of moorland roads in the Netherlands. The test vehicle was a modified Volvo 245 station wagon.	"More important, the SD of the lateral position, which reflects swerving behaviour, is lower on the experimental (narrow) sections" (Section 3.1 of De Waard et el., 1995 [4]).

Supplementary Table. 3: Literature for metric 2 of the road scenario-lane width

		Paper	On-road/ Simulator	Description	Conclusion
2. Lane width		The effect of lane width on speed (metric 2) was validated using the driving simulator based study of Liu et al., 2016 [6]. The simulator has a motion platform and has been validated for use in research. We also cite the on-road study of Fitzpatrick et al., 2000 [7] who found similar results that validate the DRF model's predictions. However, we used the simulator based study of Liu et al., 2016 [6] since the conditions they used (lane widths) were similar to those simulated for the DRF model.			
	Metric 2	Liu et al., 2016 [6]	Simulator	(N=24) The driving simulator of Tongji University - China is a motion-base simulator. A real car is placed in the middle of the experimental cabin as the test vehicle. This simulator has been validated in published literature ([8][9]).	Speed increased with lane width (Main text: Fig. 4-2d).
		Fitzpatrick et al., 2000 [7]	On-road	(N=100) In this on-road study, the speed data was collected between April 1998 and June 1999 during daylight, off-peak periods, and under dry weather conditions. Speed profiles for approximately 100 free-flowing vehicles were taken at each site (several sites in Texas, USA). Vehicle type was identified by observation. The speed profiles were collected using laser guns positioned on the side of the roadway. Techniques used to hide the technicians from passing motorists include the truck blind and locating behind a tree or bushes were used.	Speed increased with lane width (Fig. 8-6 of Fitzpatrick et al., 2000 [7]).

Supplementary Table. 4: Literature for metrics 1 and 2 of the road scenario-on road obstacles

		Paper	On-road/ Simulator	Description	Conclusion
3. On-road obstacles		The effect of on-road obstacles (parked cars on the road) was validated using the simulator study of Edquist et al., 2012 [10]. Results similar to that predicted by the DRF model and Edquist et al., 2012 [10], in terms of speed adaptation are also found in the on-road study of Daisa & Peers, 1997 [11]. We chose the simulator study of Edquist et al., 2012 [10] over Daisa & Peers, 1997 [11] because it examined both speed and lateral position effect of parked cars.			
	Metric 1 & 2	Edquist et al., 2012 [10]	Simulator	(N=29) The experiment used an EF-X by Eca-Faros driving simulator with modified software for research purposes. Twenty-nine drivers (15 male) with an average number of years participants had been driving was 9.8 (SD = 8.0); all participants were regular drivers with at least one year of licensed driving experience.	The mean lateral position of the vehicles shifted away from the parked cars (Main text: Fig. 4-3c). The mean speed reduced in the presence of parked cars (Main text: Fig. 4-3f)
		Ivan et al., 2009 [12]	On-road	(N=6900) Two sections of the same road about a mile apart in the same town (Rte. 77 in Guilford). The pavement widths are different, as one location has on-street parking but the other doesn't. However, the pavement width at the site without parking is about the same as the width of the travel lanes at the site with parking.	Speed decreased with increase in parking density (Fig. 6-3 of Ivan et al., 2009 [12]).

Supplementary Table. 5: Literature for metrics 1 and 2 of the road scenario-roadside furniture

		Paper	On-road/ Simulator	Description	Conclusion
4. Roadside furniture		The effect of roadside furniture was validated using the simulator study of Dunning et al., 2015 [13]. Results similar to that predicted by the DRF model and Dunning et al., 2015 [13], in terms of speed and lateral deviation adaptation are also found in another simulator study of Calvi, 2015 [14]. We chose the simulator study of Dunning et al., 2015 [13] over Calvi, 2015 [14] because it examined both symmetric and asymmetric roadside furniture. Calvi, 2015 [14] only examined asymmetric roadside furniture (Trees on one side and oncoming lane on the other).			
	Metric 1 & 2	Dunning et al., 2015 [13]	Simulator	(N=12) In the experiment, subjects maintained one-dimensional "steering" control of a vehicle in a driving simulation. The goal of the game was to complete each trial as quickly as possible, where the speed of the car was determined solely by position on a two-lane road.	Lateral position of the participants shifted towards the less dangerous grass in the asymmetric case and remained in the center in the symmetric case (Main text: Fig. 4-4c). Participants, on average, drove slower in the symmetric case (Main text: Fig. 4-4f).
		Calvi, 2015 [14]	Simulator	(N=44) The experiments were performed with the fixed-base CRISS driving simulator at Roma Tre University. The apparatus consisted of a real car with a force-feedback steering wheel, brake pedal, and accelerator. The system was widely validated in previous studies ([15] [16]) and used for evaluating driving performance in terms of speed, acceleration, and trajectory under various driving conditions and road environments.	"When trees were closer, drivers saw the trees as a risk, slowed down, and moved further away from them." (Abstract and Table 2 of Calvi, 2015 [14]).

Supplementary Table. 6: Literature for metrics 1 and 2 of the traffic scenario-car following

		Paper	On-road/ Simulator	Description	Conclusion
5. Car following	The effect of car following on Time Headway (THW: metric 1) was validated using the on-road study of He et al., 2002 [17]. The effect on acceleration at braking point (metric 2) was validated using the on-road study of Van der Horst, 2004 [18].				
	Metric 1	He et al., 2002 [17]	On-road	(N=184,546) Data in both constrained and free flow traffic were collected. By using a set of traffic flow measuring apparatus based on switch sensors, more than 196,000 field data, with flow varying from 50 to 1900 veh/h/lane, had been collected at JingShi, Jinghua and Guang Fuo highway, located at Beijing and Guangdong province, China. After data validation process, 184,546 pairs data of vehicle pairs can be used in the study, only 5.87% of data was eliminated.	The preferred time headway under steady-state car following (THW_{pref}) is almost constant and independent of the lead car speed (Main text: Fig. 5-1c).
	Metric 2	Van der Horst, 2004 [18]	On-road	(N=12) The experiment was conducted on a former runway 540 m long and 60 m wide in Vancouver, Canada. Participants drove an instrumented car which approached a Styrofoam model of a car. The instrumented car used in the experiment was a front wheel drive Dodge Mini Ram.	Braking intensity (deceleration at the onset of braking) increased as the approach-speed increased (Main text: Fig. 5-1f).

Supplementary Table. 7: Literature for metrics 1 and 2 of the traffic scenario-overtaking

		Paper	On-road/ Simulator	Description	Conclusion
6. Overtaking	The effect of overtaking on overtake distance (metric 1) was validated using the on-road study of Crawford, 2007 [19]. The effect on time to collision (TTC) at lane change (metric 2) was validated using the on-road study of Chen et al., 2005 [20].				
	Metric 1	Crawford, 2007 [19]	On-road	(N=8) The experiment was carried out on a straight two-lane road 22 ft wide laid out along the edge of an airfield runway 2000 yd long. Participants drove a 2.25 liter (engine displacement) saloon car.	Overtake distance increased with the speed of the overtaken car (Main text: Fig. 5-2c).
	Metric 2	Chen et al.,, 2015 [20]	On-road	(N=45) The 100-Car study was a landmark large-scale naturalistic driving study (NDS) conducted by the Virginia Tech Transportation Institute-USA from 2001 to 2004. A total of 46,250 trips from 45 drivers were used in this study. A total of 326,238 lane changes were found in the 46,250 trips. The distribution of left side and right side lane changes was essentially even.	TTC at (start of) lane change increased with the speed of the overtaken car (Main text: Fig. 5-2f).

Supplementary Table. 8: Literature for metrics 1 and 2 of the road scenario-oncoming traffic

		Paper	On-road/ Simulator	Description	Conclusion
7. Oncoming traffic	The effect of oncoming traffic on lateral deviation away from the oncoming traffic (metric 1) and speed (metric 2) was validated using the on-road study of Rasanen., 2005 [21].				
	Metric 1 & 2	Rasanen, 2005 [21]	On-road	(N=6599) This study was conducted along a curve on a two-lane road section of main road 4 in Finland where the barrier line was worn out due to encroachment by cars. The length of the left turning curve was 467 m, radius of curvature 990 m and speed limit 100 km/h.	Lateral position of ego car moves away from the oncoming traffic (Main text: Fig. 5-3c). No significant difference in the speed between the oncoming traffic 'absent' and 'present' conditions (Main text: Fig. 5-3f).

As it can been, 6 out of the 7 scenarios have results from on-road/ test track based studies from published literature that support the predictions of the DRF model. Also, the simulator based studies used for validation are mostly performed in previously validated high-fidelity simulators. Hence we think that the results found in these studies are valid indications of on-road human driving behaviour.

Supplementary Notes

These notes explain which figures/ data, from the corresponding literature, were used and the unit conversions (for example, km h^{-1} to m s^{-1}) that we performed.

Road scenarios

- **Supplementary Note 1 - Main text Fig. 4-1b. Xu et al. (2018) [1]** This figure is adopted from Fig. 10 on Page 13
- **Supplementary Note 2 - Main text Fig. 4-1d. Taragin & Leisch (1954) [2]:** This figure is created from the data of Taragin & Leisch (1954) [2] and adopted from Fig. 9 of McLean (1974) [22]. That figure contains data points from Taragin & Leisch (1954) [2] and the Department of Main Roads-NSW, (1969) [23]. We only plot the data points from Taragin & Leisch (1954) [2].
- **Supplementary Note 3 - Main text Fig. 4-2b. Godley et al. (2004) [3]:** This figure is adopted from Fig. 5. We arranged the lane widths in the ascending order, while in the original figure it is in the descending order.
- **Supplementary Note 4 - Main text Fig. 4-2d. Liu et al. (2016) [6]:** This figure shows the data from Table. 2. We only show speed for 0.5m shoulder width right lane.
- **Supplementary Note 5 - Main text Fig. 4-3c. Edquist et al. (2012) [10]:** This figure is adopted from Fig. 4 of the corresponding paper. We reversed the direction of the Y axis since in the original plot negative values of lateral deviation from lane centre indicate to the left of the lane centre. In the DRF model's convention (which is maintained consistent throughout the paper). We plot only the 'No lead vehicle' case, since that's the most relevant to our discussion.
- **Supplementary Note 6 - Main text Fig. 4-3f. Edquist et al. (2012) [10]:** This figure is adopted from Fig. 2 of the corresponding paper. We only plot the 'Mean speed' condition for empty and full parking, since that's the most relevant for our discussion.
- **Supplementary Note 7 - Main text Fig. 4-4c. Dunning et al. (2015) [13]:** This figure was adopted from Fig. 3 of the corresponding paper. Out of the 13 lateral position distribution figures we only show 2 plots. The plots for 'Standard Deviation of Motor Noise = 8' for 'Symmetric-High cost' and 'Asymmetric case'.
- **Supplementary Note 8 - Main text Fig. 4-4f. Dunning et al. (2015) [13]:** This figure was adopted from Fig. 5 of the corresponding paper. Out of the 13 box plots we only show 2 plots. The plots for 'Standard Deviation of Simulated Motor Noise = 8' for 'Symmetric-High cost' and 'Asymmetric case'.

Traffic scenarios

- **Supplementary Note 9 - Main text Fig. 5-1c. He et al. (2002) [17]:** This figure is adopted from Fig. 3. The X axis is plotted in m s^{-1} rather than Km h^{-1}
- **Supplementary Note 10 - Main text Fig. 5-1f. Van der Horst (2004) [18]:** This figure is adopted from Fig. 3 (the bottom subplot). We only plot the 'no occlusion' case, since the DRF model did not simulate any occluded conditions.
- **Supplementary Note 11 - Main text Fig. 5-2c. Crawford (2007) [19]:** This figure is adopted from Fig. 5. We plotted only the 'Average overtaking distance' condition and omitted the 'Average of each driver's best performance' condition.
- **Supplementary Note 12 - Main text Fig. 5-2f. Chen et al. (2015) [20]:** This figure is adopted Fig. 3. We plot all the points and the 50 percentile line. We did not plot the 10 percentile line.

- **Supplementary Note 13 - Main text Fig. 5-3c. Ransen (2005) [21]:** This figure is plotted from Table 3 of the corresponding paper. We plotted the data of only the 'Before' condition (which refers to the road before lane dividing measures were taken) for passenger cars. The DRF model also did not simulate a lane divider. To convert the original data (say x) provided in the table into the same convention as used for DRF model (say y), we took the following steps:
  1. Convert cm to m : x1=x /100
  2. Ransen (2005) [21] measured the lateral distance from lane edge to left tyre. Hence we assumed an average vehicle width of 1.8m : x2= x1+1.8/2
  3. To state the distance from lane centre we subtracted the (lane width)/2 : x3=x2-3.4/2 (Lane width they used was 3.4m)
  4. To make left of lane centre means positive lateral deviation values multiplied by -1 : x4=-x3
  5. Data plotted in Results figure of DRF model : y=x4
- **Supplementary Note 14 - Main text Fig. 5-3f. Rasanen (2005) [21]:** This figure is plotted from Table 3 of the corresponding paper. We plotted the data of only the 'Before' condition (which refers to the road before lane dividing measures were taken) for passenger cars. The DRF model also did not simulate a lane divider. km h^{-1} was converted to m s^{-1}.

References

[1] Xu, J., Luo, X. & Shao, Y.-M. Vehicle trajectory at curved sections of two-lane mountain roads: a field study under natural driving conditions. *European Transport Research Review* **10**, 12 (2018).

[2] Taragin, A. & Leisch, L. Driver performance on horizontal curves. In *Highway Research Board Proceedings*, vol. 33 (1954).

[3] Godley, S. T., Triggs, T. J. & Fildes, B. N. Perceptual lane width, wide perceptual road centre markings and driving speeds. *Ergonomics* **47**, 237–256 (2004).

[4] Waard, D. D., Jessurun, M., Steyvers, F. J., Reggatt, P. T. & Brookhuis, K. A. Effect of road layout and road environment on driving performance, drivers' physiology and road appreciation. *Ergonomics* **38**, 1395–1407 (1995).

[5] Godley, S. T., Triggs, T. J. & Fildes, B. N. Driving simulator validation for speed research. *Accident analysis & prevention* **34**, 589–600 (2002).

[6] Liu, S., Wang, J. & Fu, T. Effects of lane width, lane position and edge shoulder width on driving behavior in underground urban expressways: a driving simulator study. *International Journal of Environmental Research and Public Health* **13**, 1010 (2016).

[7] Fitzpatrick, K., Carlson, P. J., Wooldridge, M. D. & Brewer, M. A. Design factors that affect driver speed on suburban arterials. Tech. Rep. (2000).

[8] Chen, Y. & Zheng, S. Mechanism analysis of vehicles operating characteristic affected by visual environment of underground road. *Journal of Tongji University (Natural Science)* 13 (2013).

[9] Changbin, C., Junhua, W. & Yangming, L. Driving simulator validation for research on driving behavior at entrance of urban underground road. In *2015 International Conference on Transportation Information and Safety (ICTIS)*, 147–150 (IEEE, 2015).

[10] Edquist, J., Rudin-Brown, C. M. & Lenné, M. G. The effects of on-street parking and road environment visual complexity on travel speed and reaction time. *Accident Analysis & Prevention* **45**, 759–765 (2012).

[11] Daisa, J. M. & Peers, J. Narrow residential streets: do they really slow down speeds? In *Institute of Transportation Engineers 67th annual Meeting Institute of Transportation Engineers (ITE)* (1997).

[12] Ivan, J. N., Garrick, N. W. & Hanson, G. Designing roads that guide drivers to choose safer speeds. Tech. Rep. (2009).

[13] Dunning, A., Ghoreyshi, A., Bertucco, M. & Sanger, T. D. The tuning of human motor response to risk in a dynamic environment task. *PloS one* **10** (2015).

[14] Calvi, A. Does roadside vegetation affect driving performance?: Driving simulator study on the effects of trees on drivers' speed and lateral position. *Transportation Research Record* **2518**, 1–8 (2015).

[15] Bella, F. Validation of a driving simulator for work zone design. *Transportation Research Record* **1937**, 136–144 (2005).

[16] Bella, F. Driving simulator for speed research on two-lane rural roads. *Accident Analysis & Prevention* **40**, 1078–1087 (2008).

[17] He, M., Liu, X. & Rong, J. Driver's preferred time headway selection: Experimental findings. In *Traffic And Transportation Studies (2002)*, 889–894 (2002).

[18] Van der Horst, R. Occlusion as a measure for visual workload: an overview of tno occlusion research in car driving. *Applied Ergonomics* **35**, 189–196 (2004).

[19] Crawford, A. The overtaking driver. *Ergonomics* **6**, 153–170 (1963).

[20] Chen, R., Kusano, K. D. & Gabler, H. C. Driver behavior during overtaking maneuvers from the 100-car naturalistic driving study. *Traffic Injury Prevention* **16**, S176–S181 (2015).

[21] Räsänen, M. Effects of a rumble strip barrier line on lane keeping in a curve. *Accident Analysis & Prevention* **37**, 575–581 (2005).

[22] McLean, J. Driver behaviour on curves-a review. In *Australian Road Research Board (ARRB) Conference, 7th, 1974, Adelaide*, vol. 7 (1974).

[23] Department of Main Roads, N. S. W. The behaviour of drivers on horizontal curves. *Main Roads* **34**, 127–128 (1969).

[24] Kolekar, S., De Winter, J. & Abbink, D. Which parts of the road guide obstacle avoidance? quantifying the driver's risk field. *Applied Ergonomics* **89**, 103196 (2020).
