# Supplementary material for: Human-like driving behaviour emerges from a risk-based driver model
Source: Nat Commun. 2020 Sep 29;11:4850. doi: 10.1038/s41467-020-18353-4 (PMC7525534; doi:10.1038/s41467-020-18353-4)
Supplement: Supplementary file 3 — Description of Additional Supplementary Files [file 41467_2020_18353_MOESM3_ESM.pdf]

Description of Additional Supplementary Files

File Name: Supplementary Movie 1
Description: This video shows the track and scenarios implemented in the fixed base driving simulator.

File Name: Supplementary Movie 2
Description: This videos shows the simulation of the DRF model with ’normal’ parameter settings.

File Name: Supplementary Movie 3
Description: This videos shows the simulation of the DRF model with ’sport’ parameter settings.
