# Supplementary material for: Human-like driving behaviour emerges from a risk-based driver model
Source: Nat Commun. 2020 Sep 29;11:4850. doi: 10.1038/s41467-020-18353-4 (PMC7525534; doi:10.1038/s41467-020-18353-4)
Supplement: Supplementary file 7 — Reporting Summary [file 41467_2020_18353_MOESM7_ESM.pdf]

nature research

Corresponding author(s): Sarvesh Kolekar
Last updated by author(s): Jul 27, 2020

Reporting Summary

Nature Research wishes to improve the reproducibility of the work that we publish. This form provides structure for consistency and transparency in reporting. For further information on Nature Research policies, see our Editorial Policies and the Editorial Policy Checklist.

Statistics

For all statistical analyses, confirm that the following items are present in the figure legend, table legend, main text, or Methods section.

n/a	Confirmed	
☐	☒	The exact sample size (n) for each experimental group/condition, given as a discrete number and unit of measurement
☒	☐	A statement on whether measurements were taken from distinct samples or whether the same sample was measured repeatedly
☒	☐	The statistical test(s) used AND whether they are one- or two-sided<br>*Only common tests should be described solely by name; describe more complex techniques in the Methods section.*
☒	☐	A description of all covariates tested
☒	☐	A description of any assumptions or corrections, such as tests of normality and adjustment for multiple comparisons
☒	☐	A full description of the statistical parameters including central tendency (e.g. means) or other basic estimates (e.g. regression coefficient) AND variation (e.g. standard deviation) or associated estimates of uncertainty (e.g. confidence intervals)
☒	☐	For null hypothesis testing, the test statistic (e.g. F, t, r) with confidence intervals, effect sizes, degrees of freedom and P value noted<br>*Give P values as exact values whenever suitable.*
☒	☐	For Bayesian analysis, information on the choice of priors and Markov chain Monte Carlo settings
☒	☐	For hierarchical and complex designs, identification of the appropriate level for tests and full reporting of outcomes
☒	☐	Estimates of effect sizes (e.g. Cohen's d, Pearson's r), indicating how they were calculated

Our web collection on statistics for biologists contains articles on many of the points above.

Software and code

Policy information about availability of computer code

Data collection	The experiment was conducted in a fixed based driving simulator at TU Delft, Netherlands. This simulator uses DUECA: DUECA, Delft University Environment for Communication and Activation. It is a middleware layer for the implementation and deployment of real-time simulations (or other computational processes) on distributed computing hardware.
Data analysis	A custom code was written in Matlab 2016b on a windows10 OS. The code has been made available and the doi is provided in the 'Data availability' and 'Code availability' sections.

For manuscripts utilizing custom algorithms or software that are central to the research but not yet described in published literature, software must be made available to editors and reviewers. We strongly encourage code deposition in a community repository (e.g. GitHub). See the Nature Research guidelines for submitting code & software for further information.

Data

Policy information about availability of data

All manuscripts must include a data availability statement. This statement should provide the following information, where applicable:

- Accession codes, unique identifiers, or web links for publicly available datasets
- A list of figures that have associated raw data
- A description of any restrictions on data availability

The Driving simulator experiment data, the simulation data that support the findings of this study, and the source data for figures 3,4, and 5 are available in the 4TU.Centre for Research Data with the identifier: https://doi.org/10.4121/uuid:8132bccd-e900-4ba0-942e-c3114502bda2

Field-specific reporting

Please select the one below that is the best fit for your research. If you are not sure, read the appropriate sections before making your selection.

☐ Life sciences ☒ Behavioural & social sciences ☐ Ecological, evolutionary & environmental sciences

For a reference copy of the document with all sections, see nature.com/documents/nr-reporting-summary-flat.pdf

Behavioural & social sciences study design

All studies must disclose on these points even when the disclosure is negative.

Study description	The study models human driving behaviour. It is based on quantitative data obtained from human experiment in a driving simulator and based on previously published literature. This data is then compared to the predictions made by the model.
Research sample	In this study, parameter estimation for the model was done using one 25 year old male participant, who was an employee at TU Delft, Netherlands. However, this study uses data from previously published literature. It validates the model based on results that were found by several researchers, in several different studies. Each of the papers used for literature use different population sizes and demographics. The sample size is mentioned in the Supplementary Tables 1-8. For all other details of the experiments, we will have to refer to the corresponding research paper.
Sampling strategy	We had only 1 participant. This was sufficient because the data was used to estimate realistic parameters for the model. The actual validation of the model was done by comparison with literature. Each paper used different sampling strategies, which will have to be referred to the corresponding papers.
Data collection	The volunteer drove ten times in a fixed base driving simulator, with the instruction, “drive as you normally would” and ten times with, “drive faster”. The researcher was sitting in a room outside the simulator. The data was collected using the computers of the simulator.
Timing	The participant came on 2 separate days: 2019-05-01 and 2019-05-06. On the 1st day he performed 10 trials of 'normal' driving and on 2nd day the participant performed 10 trials of 'sport' driving.
Data exclusions	No exclusions
Non-participation	No dropouts
Randomization	We decided to ask the participant to perform all 10 trials of 'normal' driving on one day and all 10 trials of 'sport' driving on the 2nd day (5 days apart) because, we then expect the participant would show consistent/ steady state 'normal' or 'sport' behaviour.

Reporting for specific materials, systems and methods

We require information from authors about some types of materials, experimental systems and methods used in many studies. Here, indicate whether each material, system or method listed is relevant to your study. If you are not sure if a list item applies to your research, read the appropriate section before selecting a response.

Materials & experimental systems

n/a	Involved in the study	
☒	☐	Antibodies
☒	☐	Eukaryotic cell lines
☒	☐	Palaeontology and archaeology
☒	☐	Animals and other organisms
☐	☒	Human research participants
☒	☐	Clinical data
☒	☐	Dual use research of concern

Methods

n/a	Involved in the study	
☒	☐	ChIP-seq
☒	☐	Flow cytometry
☒	☐	MRI-based neuroimaging

Human research participants

Policy information about studies involving human research participants

Population characteristics	(N=1) A 25-year-old male volunteer drove ten times with the instruction, “drive as you normally would” and ten times with, “drive faster”. This was meant to emulate ‘normal’ and ‘sport’ driving behaviour. This data was used only to perform parameter estimation for the model.<br>The actual validation of the model was done using data from literature, where different papers had different population characteristic. The number of participants for each of the validation study can be found in the Supplementary Tables 1-8. All the other details will have to be gathered from the corresponding paper.
Recruitment	The recruitment was done via email.
Ethics oversight	Human Research Ethics Committee (HREC)-TU Delft, the Netherlands.

Note that full information on the approval of the study protocol must also be provided in the manuscript.
